# Multi-region exome sequencing reveals genomic evolution from preneoplasia to lung adenocarcinoma

**DOI:** 10.1038/s41467-019-10877-8

**Published:** 2019-07-05

**Authors:** Xin Hu, Junya Fujimoto, Lisha Ying, Junya Fukuoka, Kazuto Ashizawa, Wenyong Sun, Alexandre Reuben, Chi-Wan Chow, Nicholas McGranahan, Runzhe Chen, Jinlin Hu, Myrna C. Godoy, Kazuhiro Tabata, Kishio Kuroda, Lei Shi, Jun Li, Carmen Behrens, Edwin Roger Parra, Latasha D. Little, Curtis Gumbs, Xizeng Mao, Xingzhi Song, Samantha Tippen, Rebecca L. Thornton, Humam Kadara, Paul Scheet, Emily Roarty, Edwin Justin Ostrin, Xu Wang, Brett W. Carter, Mara B. Antonoff, Jianhua Zhang, Ara A. Vaporciyan, Harvey Pass, Stephen G. Swisher, John V. Heymach, J. Jack Lee, Ignacio I. Wistuba, Waun Ki Hong, P. Andrew Futreal, Dan Su, Jianjun Zhang

**Affiliations:** 1 Department of Genomic Medicine, The University of Texas MD Anderson Cancer Center, Houston, TX 77030 USA; 2 Department of Translational Molecular Pathology, The University of Texas MD Anderson Cancer Center, Houston, TX 77030 USA; 3 Institute of Cancer Research and Basic Medical Sciences of Chinese Academy of Sciences, Cancer Hospital of University of Chinese Academy of Sciences, Zhejiang Cancer Hospital & Key Laboratory Diagnosis and Treatment Technology on Thoracic Oncology of Zhejiang Province, 310022 Hangzhou, China; 4 Department of Pathology, Nagasaki University Graduate School of Biomedical Sciences, 8528523 Nagasaki, Japan; 5 Department of Clinical Oncology, Nagasaki University Graduate School of Biomedical Sciences, 8528523 Nagasaki, Japan; 6 Department of Pathology, Institute of Cancer Research and Basic Medical Sciences of Chinese Academy of Sciences, Cancer Hospital of University of Chinese Academy of Sciences, Zhejiang Cancer Hospital, 310022 Hangzhou, China; 7 Department of Thoracic/Head and Neck Medical Oncology, The University of Texas MD Anderson Cancer Center, Houston, TX 77030 USA; 8 Cancer Research United Kingdom-University College London Lung Cancer Centre of Excellence, London, WC1E6BT UK; 9 Department of Diagnostic Radiology, The University of Texas MD Anderson Cancer Center, Houston, TX 77030 USA; 10 Department of Radiology, Institute of Cancer Research and Basic Medical Sciences of Chinese Academy of Sciences, Cancer Hospital of University of Chinese Academy of Sciences, Zhejiang Cancer Hospital, 310022 Hangzhou, China; 11 Department of Epidemiology, The University of Texas MD Anderson Cancer Center, Houston, TX 77030 USA; 12 Department of General Internal Medicine, The University of Texas MD Anderson Cancer Center, Houston, TX 77030 USA; 13 Department of Thoracic and Cardiovascular Surgery, The University of Texas MD Anderson Cancer Center, Houston, TX 77030 USA; 14 Department of Cardiothoracic Surgery, New York University Langone Medical Center, New York, NY 10016 USA; 15 Department of Biostatistics, The University of Texas MD Anderson Cancer Center, Houston, TX 77030 USA

**Keywords:** Cancer genomics, Cancer genomics, Non-small-cell lung cancer, Non-small-cell lung cancer

## Abstract

There has been a dramatic increase in the detection of lung nodules, many of which are preneoplasia atypical adenomatous hyperplasia (AAH), adenocarcinoma in situ (AIS), minimally invasive adenocarcinoma (MIA) or invasive adenocarcinoma (ADC). The molecular landscape and the evolutionary trajectory of lung preneoplasia have not been well defined. Here, we perform multi-region exome sequencing of 116 resected lung nodules including AAH (n = 22), AIS (n = 27), MIA (n = 54) and synchronous ADC (n = 13). Comparing AAH to AIS, MIA and ADC, we observe progressive genomic evolution at the single nucleotide level and demarcated evolution at the chromosomal level supporting the early lung carcinogenesis model from AAH to AIS, MIA and ADC. Subclonal analyses reveal a higher proportion of clonal mutations in AIS/MIA/ADC than AAH suggesting neoplastic transformation of lung preneoplasia is predominantly associated with a selective sweep of unfit subclones. Analysis of multifocal pulmonary nodules from the same patients reveal evidence of convergent evolution.

## Abstract (web summary)

There has been a drastic increase in detection of lung nodules, many of which are precancers, preinvasive, minimally invasive or sometimes invasive lung cancers. Here, Hu et al. perform multi-region exome sequencing to discern the evolutional trajectory from precancers to invasive lung cancers.

## Introduction

Lung cancer is potentially curable when detected early as demonstrated in the National Lung Cancer Screening Trial^1^. The growing implementation of lung cancer screening and advent of high-resolution computed tomography (CT) for diagnostic imaging have resulted in a dramatic increase in the detection of indeterminate pulmonary nodules (IPNs). Many IPNs are atypical adenomatous hyperplasia (AAH), preinvasive adenocarcinoma in situ (AIS), minimally invasive adenocarcinoma (MIA), or sometimes early invasive lung adenocarcinoma (ADC)^2–5^. It has been postulated that AAH, the only recognized preneoplasia to ADC, may progress to AIS, MIA, and eventually frankly invasive ADC^6^. However, the molecular landscape of these lesions has not been well defined and the evolutionary trajectory from AAH to ADC remains controversial.

Carcinogenesis of lung cancer may result from accumulation of mutations in a branched evolutionary model like a growing tree^7–10^, where the trunk harbors early founder events, while the branches represent subsequent events acquired later during carcinogenesis. Multi-region sequencing can depict genomic events to their relative molecular time with early events ubiquitously present in every tumor region and late events confined to spatially separated tumor regions. Using this approach, we have previously delineated the genomic evolution of localized non-small cell lung cancers (NSCLC) and demonstrated that a majority of canonical cancer gene mutations were early events during lung carcinogenesis^11–13^, suggesting that comprehensive molecular profiling of preneoplasia is warranted to fully understand the molecular evolution during the initiation of lung cancer.

Over the past decade, genome-wide profiling has substantially advanced our understanding of the genomic landscapes of various cancer types and led to the identification of novel predictive/prognostic biomarkers and therapeutic targets^14–17^. However, the comprehensive genomic landscape of lung preneoplasia, preinvasive, and early invasive lung cancer has not been well studied, primarily due to the scarcity of resected specimens, as surgery is not the standard of care for the management of IPNs.

To delineate the pivotal molecular events driving lung cancer initiation and early progression, we initiated an international collaboration to collect and characterize a large cohort of resected IPNs. Herein, we report the analyses of multi-region whole-exome sequencing (WES) of 116 resected IPNs including AAH (*N* = 22), AIS (*N* = 27), MIA (*N* = 54), and synchronous invasive ADC (*N* = 13) from 53 patients (Supplementary Data 1). Our results demonstrate evidence of genomic evolution from AAH to AIS, MIA, and ADC, and suggest that neoplastic transformation of lung preneoplasia may be predominantly associated with selective sweep of unfit subclones.

## Results

### Multi-region exome sequencing of resected pulmonary nodules

In this cohort, 11 patients were from China and 42 were from Japan, and there were 25 smokers and 28 non-smokers (Supplementary Data 1). In total, 267 multi-region samples were subjected to WES with a mean sequencing depth of 150×. Matched DNA from normal lung tissue (≥2 cm from tumor margin, morphologically negative for malignant cells assessed by two lung cancer pathologists independently) was used as germ line DNA control. A total of 46,007 somatic single nucleotide variants (SNVs) were identified (Supplementary Data 2).

### Quality control for sequencing data from FFPE samples

As all specimens were formalin-fixed paraffin-embedded (FFPE) samples, which are known to be associated with sequencing artifacts, rigorous quality control was applied before further analyses. FFPE artifacts usually present as non-recurrent, low log odds (LOD) score, low variant allelic frequency (VAF) (usually <10%), predominantly C > T/G > A “transitions”^18^. Therefore, in addition to sequencing depth, VAF and minimal counts of alternative reads, a minimal LOD threshold of 10 (the default is 6.3 for somatic mutation calls) was applied to filter out FFPE artifacts. As shown in Supplementary Fig. 2, “mutations” with low LOD scores exhibited high proportion of C > T/G > A transitions, while mutations with high LOD scores showed consistent proportion of C > T/G > A transitions, suggesting “mutations” with low LOD scores were likely enriched for FFPE artifacts.

We then assessed the quality of mutation calls after our stringent filtering by comparing the proportion of C > T/G > A transitions in mutations with VAF < 10% versus mutations with VAF > 10%. Overall, 26.8% of mutations with VAF < 10% were C > T/G > A transitions versus 22.1% for mutations with VAF > 10% for this cohort of IPNs. These proportions are comparable to the high quality frozen samples from TCGA lung adenocarcinoma (LUAD) cohort, where 32.2% of mutations with VAF < 10% were C > T/G > A transitions versus 24.3% for mutations with VAF > 10%. We also scrutinized each sample for an excess of C > T/G > A. As shown in Supplementary Data 3, there were 12 out of the 267 (4.5%) samples having C > T/G > A transitions over 56.25%, the cutoff for top 5% of TCGA LUAD samples with high C > T/G > A transitions. Importantly, all 12 samples had low mutation burden with 10 of 12 having 25 mutations or less, which made the estimation of mutation spectrum less reliable. In addition, 8 of the 12 samples were from never smokers, which are known to be associated with C > T/G > A predominant mutational spectra. Taken together, these data suggest FFPE artifacts were controlled for the current study.

### Progressive evolution of AAH at single nucleotide level

We next delineated the genomic landscape of these IPNs at single nucleotide level. The SNV profiles varied substantially between IPNs of different histologic stages as well as between IPNs of the same histologic stages, highlighting substantial inter-patient heterogeneity. Overall, the total mutational burden (TMB) progressively increased from AAH to AIS, further to MIA and ADC (Supplementary Fig. 1a–b and Supplementary Fig. 3), suggesting a progressive accumulation of SNVs along with early neoplastic evolution. To more conservatively rule out any impact of potential FFPE artifacts on TMB, we recalculated TMB by removing all private subclonal mutations detected in only one region of any given IPNs or by removing all C > T/G > A transitions. As shown in Supplementary Fig. 4, the patterns of TMB remained the same.

**Fig. 1:**
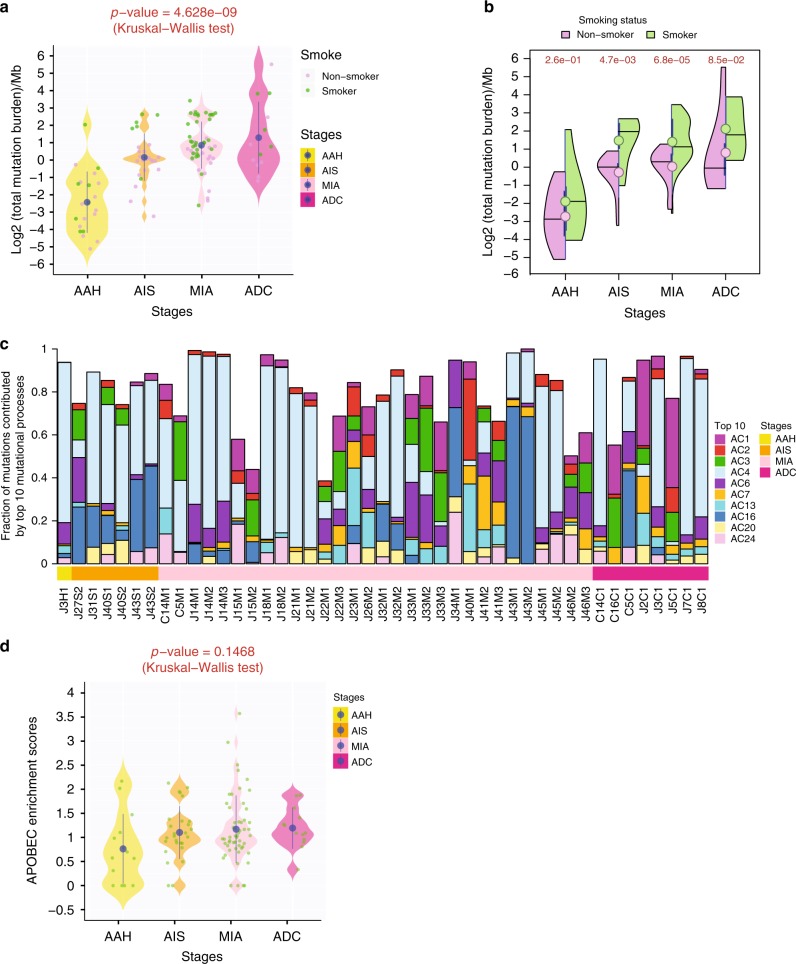
Progressive genomic evolution from AAH to ADC at the single nucleotide level. **a** Mutational burden. Each dot represents the mutational burden in each IPN from smokers (green) or non-smokers (purple). The solid blue dots represent the mean mutational burden of all lesions of each histologic stage. Kruskal–Wallis H test was used to compare mutational burden among all stages. **b** Mutational burden in smokers versus non-smokers. The violin plots represent the distribution of mutational burden in smokers (green) and non-smokers (purple), respectively, by each stage. The circles represent the mean mutational burden of IPNs from smokers (green) or non-smokers (purple) by each stage. Wilcoxon Rank-Sum test was used for the comparison between smokers and non-smokers. **c** Top 10 enriched mutational signatures. The Alexandrov-COSMIC mutational signatures were derived from all mutations in each IPN. Only IPNs with a minimum of 100 unique SNVs were included in mutational signature deconstruction. The stacked bar plot represents the fraction of estimated mutations for each signature in each IPN. **d** The enrichment of APOBEC-mediated processes. Each green dot represents APOBEC enrichment score in each IPN and the solid blue dots represent the mean APOBEC enrichment scores of all IPNs of each histologic stage with 95% confidence interval as error bars. The statistical significance between all stages was assessed by Kruskal–Wallis H test. Only lesions with a minimum of 10 SNVs were included for APOBEC enrichment analysis

To unravel the mechanisms underlying mutagenesis during initiation and progression of preneoplasia, we extracted mutational signatures derived from the patterns of somatic mutations^19^. To avoid over-fitting, we applied this analysis only to IPNs with a minimum of 100 unique SNVs. Top mutational signatures enriched in this cohort of IPNs included Alexandrov-COSMIC signature 1 (AC1, associated with spontaneous deamination), AC2 and AC13 (associated with APOBEC-mediated processes), AC3 (associated with DNA double strand break repair defect), AC4 (associated with tobacco exposure), and AC6 (associated with DNA mismatch repair defect) (Fig. 1c and Supplementary Fig. 5), indicating the potential roles of these mutational processes during early lung carcinogenesis. Recently, the APOBEC-mediated mutational processes have drawn attention because of its role in subclonal diversification during lung cancer evolution^20^ and its potential as a therapeutic target^21,22^, yet its role in the  initiation and early progression of lung preneoplasia has not been investigated. In this cohort of preneoplasia, preinvasive and early invasive lung cancers, we observed a trend of progressive increase of APOBEC-associated mutational signatures AC2 (weight score: 0.014 in AAH, 0.03005 in AIS, 0.0459 in MIA and 0.0366 in ADC) and AC13 (weight score: 0.0073 in AIS, 0.045 in MIA and 0.036 in ADC) in later-stage IPNs. To further investigate the APOBEC-mediated mutational processes during early lung carcinogenesis, we next calculated APOBEC enrichment scores^23^. APOBEC-mediated processes were observed in all four histologic stages, with a trend of more enrichment in later-stage IPNs (Fig. 1d), although the difference did not reach statistical significance, probably due to small sample size, low TMB, and substantial heterogeneity in this cohort of IPNs. These results imply an important role of the APOBEC-mediated mutational processes during initiation and progression of lung preneoplasia, with possibly increasing activities during neoplastic evolution.

### Macroevolution from AAH to ADC at chromosomal level

**Fig. 2:**
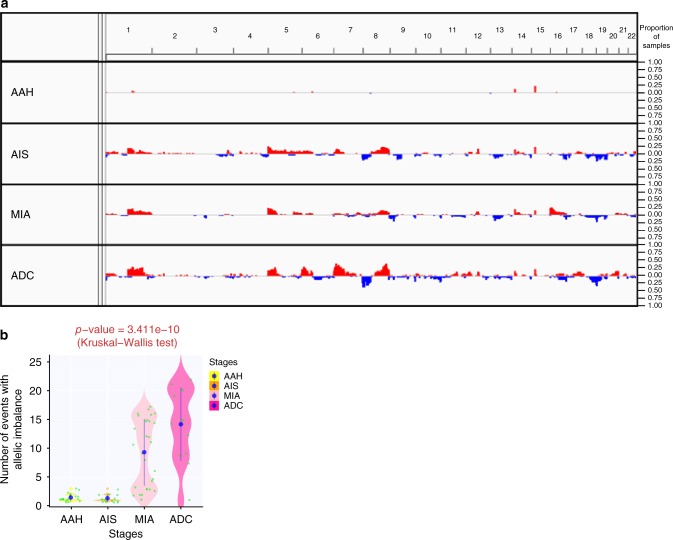
Macroevolution from AAH to ADC at chromosomal level. **a** The somatic copy number aberrations across the genome. Each row represents all lesions grouped by histologic stage. Copy number gains, defined as the mean log2 ratio (IPN versus germ line DNA) >0.3 of all lesions by each given stage, are represented as red bars. Copy number losses, defined as the mean log2 ratio (IPN versus germ line DNA) ≤0.3 of all lesions by each histologic stage are represented as blue bars. The height of the bars is proportional to the fraction of IPNs showing copy number gains or losses at corresponding chromosomal regions. **b** The allelic imbalance. Each green dot represents the number of AI events in each IPN and the blue dots represent the mean number of AI events detected in IPNs of each histologic stage. The difference between all stages was assessed by Kruskal–Wallis H test

In contrast to progressive genomic evolution from AAH to ADC at the single nucleotide level, somatic copy number alterations (SCNAs) appear to be demarcated. Very few SCNAs were detected in AAH, while SCNA events became prevalent in AIS, MIA, and ADC (Fig. 2a). GISTIC analysis identified significant (FDR *q*-value < 5e-06) chromosomal gains at 6p21.1, 12p12.1, 12q15 and chromosomal losses at 17p13.3, 19p13.3 (Supplementary Fig. 6), all of which are commonly gained and lost chromosomal regions in lung adenocarcinomas^14^. Furthermore, we estimated allelic imbalance (AI) in these IPNs using hapLOHseq^24^ and detected only a few AI events in AAH and AIS, but widespread AI events across multiple genomic regions in MIA, which further increased in ADC (*p* = 3.411e-10, Kruskal–Wallis test) (Fig. 2b and Supplementary Fig. 7). Taken together, these data imply macroevolution at the chromosomal level during the transitions from AAH to AIS and from AIS to MIA, possibly associated with SCNA and AI, respectively.

### Selective clonal sweep during neoplastic evolution of AAH

**Fig. 3:**
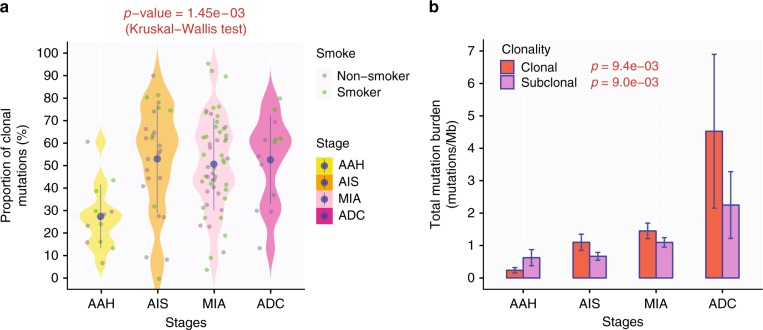
Clonal sweep from AAH to ADC. **a** Higher proportion of clonal mutations in later-stage IPNs. The mean proportions of clonal mutations in IPNs of each histologic stage are shown with 95% confidence interval as error bars. The difference between all stages was assessed by Kruskal–Wallis H test. Only IPNs with a minimum of 10 SNVs were included for subclonal analysis. **b** Progressive increase in clonal and subclonal mutations. The mean clonal mutational burden (orange) and subclonal mutational burden (purple) in AAH, AIS, MIA, and ADC are shown with 95% confidence interval as error bars. Kruskal–Wallis H test was used for comparing mutational burdens between all stages for clonal mutations and subclonal mutations

The clonal architecture of lung preneoplasia and the trajectory through which it evolves with progression from preneoplasia to invasive lung cancer are unknown. We sought to explore the clonal architecture of AAH, AIS, MIA, and ADC in our cohort. Using a modified version of pyclone^12^, we inferred clonality of somatic mutations identified in these IPNs. Overall, an average of 48.8% of all mutations (ranging from 0 to 95.3%) were clonal in these IPNs, which was significantly lower than that of invasive lung cancers (average 68.2%, ranging from 8.2 to 100%, *p* = 1.31e-10, Mann–Whitney *U* Test)^12^. Furthermore, we observed higher proportion of clonal mutations in AIS/MIA/ADC than that in AAH (Fig. 3a). Interestingly, both clonal mutational burden and subclonal mutational burden were significantly higher in later-stage IPNs (Fig. 3b). These data suggest that the progression of lung preneoplasia predominantly followed the clonal sweep model, whereby a proportion of subclonal mutations in early-stage IPNs became clonal in later-stage IPNs while unfit subclones were eliminated. However, selective sweep is typically associated with reduction in subclonal mutations in later-stage diseases, which was not observed in the current cohort. To investigate whether sequencing artifacts from FFPE samples could account for the lack of reduction in subclonal mutations in later-stage IPNs, we repeated the analysis by removing all private subclonal mutations or by removing all C > T/G > A transitions. Similar to the pattern obtained using all mutations, higher clonal mutational burden and higher subclonal mutational burden were observed in later-stage IPNs by either approach (Supplementary Fig. 8), suggesting that the lack of reduction in subclonal mutations in later-stage IPNs was unlikely due to FFPE artifacts. One plausible explanation may be that clonal sweep was accompanied by subclonal diversification in parallel, whereby a proportion of subclonal mutations become clonal with accompanying ongoing acquisition of subclonal mutations in the expanding population reflecting ongoing mutational processes.

Next, we leveraged the multi-region WES data and performed phylogenetic analysis to reconstruct the ITH architecture of each IPN. Consistent with subclonal analysis, the proportion of trunk mutations, representing genomic events acquired during early molecular time of carcinogenesis, was higher in later-stage IPNs (Supplementary Fig. 9), further supporting clonal sweeps during initiation and progression of lung preneoplasia.

### Cancer gene mutations from AAH to ADC

**Fig. 4:**
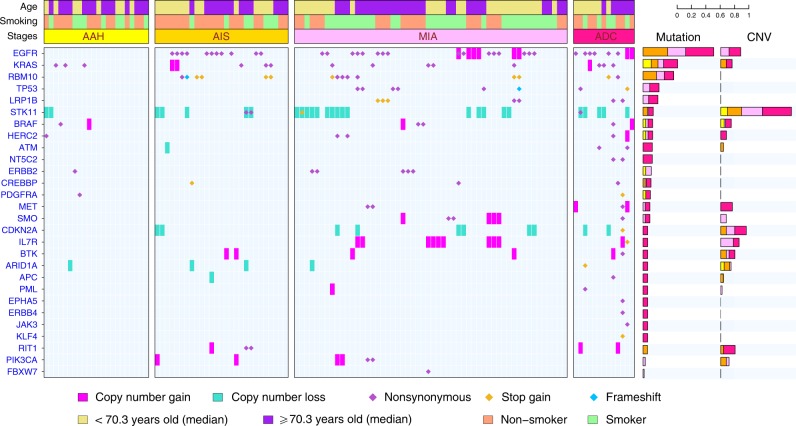
Cancer gene mutations and copy number aberrations in IPNs. Cancer gene mutations were defined as nonsynonymous mutations in known cancer genes identical to those previously reported and frame-shift indels or truncating mutations in tumor suppressor genes. Cancer genes located in chromosomal segments with copy number gains (red) or losses (green) are shown. A threshold of log2 ratio (IPN versus germ line DNA) >2 or ≤2 was used to screen for chromosomal gains or losses, respectively

Previous studies have demonstrated that a majority of canonical cancer gene mutations are early molecular events during carcinogenesis of NSCLC^11,12,25^. However, because only invasive NSCLC tumors were analyzed, these studies were unable to characterize the timing of cancer gene mutations during initiation and early progression of lung preneoplasia. Taking advantages of exome sequencing of preneoplasia and preinvasive lung cancers in the current study, we depicted the canonical cancer gene mutations and copy number variations during early carcinogenesis. As shown in Fig. 4 and Supplementary Data 4, commonly mutated cancer genes in this cohort of IPNs included *EGFR, KRAS, RBM10, TP53*, etc. In addition, *STK11* and *CDKN2A* were common tumor suppressor genes involved in chromosomal losses. *EGFR* was the most commonly mutated cancer gene in this cohort occurring in 40.7% of AIS, 29.6% of MIA and 46.2% of ADC lesions. Strikingly, no *EGFR* mutation was detected in 22 AAH lesions under our strict filtering criteria with VAF ≥ 0.05. This was different from previous studies, in which *EGFR* mutations were detected in a proportion of AAH lesions^26,27^. We therefore applied less stringent filtering criteria (VAF ≥ 0.01 and alteration reads ≥2), and observed canonical *EGFR* mutations in 7 AAH lesions (Supplementary Data 5). Comparing the cancer cell fraction (CCF) of *EGFR* mutations across different stages demonstrated that *EGFR* mutations were only present as minor subclones in AAH (mean CCF = 0.11), which became major subclones in AIS (mean CCF = 0.66), MIA (mean CCF = 0.54) and ADC (mean CCF = 0.71). Taken together, these data imply that the subclones with *EGFR* mutations may have a selective advantage and therefore became dominant clones in later-stage IPNs. In addition, loss of chromosomal segments containing *STK11* was detected in 10% of AAH, 20% of AIS, 35.7% of MIA and 38.5% of ADC lesions (*p* = 0.02558, *χ*^2^ test for trend in proportions), implying *STK11* loss may be a later genomic event during initiation or progression of lung preneoplasia. Gene expression data are needed to validate the role of *STK11* during early carcinogenesis.

### Genomic landscape of IPNs from smokers versus non-smokers

Cigarette smoking is the most important risk factor for lung cancer. It has been well documented that the genomic landscape of lung cancers from smokers is different from that of non-smokers^28^. However, whether the genomic landscapes of lung preneoplasias and preinvasive lung cancers are different between smokers and non-smokers has not been systemically studied. With the caveat of small sample size fully acknowledged, we sought to explore whether lung adenocarcinoma precursors from smokers have distinct genomic alterations and evolutionary trajectories compared to their non-smoking counterparts. As shown in Fig. 1a–b and Supplementary Fig. 10a, IPNs from smokers displayed a trend of higher TMB than those from non-smokers at all four histologic stages. In addition, smokers showed higher abundance of AI events in MIA and ADC than non-smokers, although the difference did not reach statistical significance (Supplementary Fig. 10b). Smokers also showed a trend of higher clonal TMB (Supplementary Fig. 10c), higher subclonal TMB (Supplementary Fig. 10d) and higher proportion of trunk mutations (Supplementary Fig. 10e). Although no significant difference was observed between smokers and non-smokers in regard to cancer gene mutations, likely due to small sample size, *EGFR* mutations appeared to be more frequent in non-smokers than smokers (11/28 versus 7/25, *p* = 0.283, Chi-Square test), while smokers showed higher incidence of *TP53* mutations (5/25 versus 1/28, *p* = 0.073, *χ*^2^ test) and *CDKN2A* loss or mutations (6/25 versus 2/28, *p* = 0.092, *χ*^2^ test). Taken together, these data imply that cigarette smoking may be associated with distinct evolutionary trajectories during initiation and progression of lung preneoplasia. Characterization of larger cohorts of IPNs from both smokers and non-smokers are warranted to address this critical question.

### Distinct drivers and genetic constraints in multifocal IPNs

**Fig. 5:**
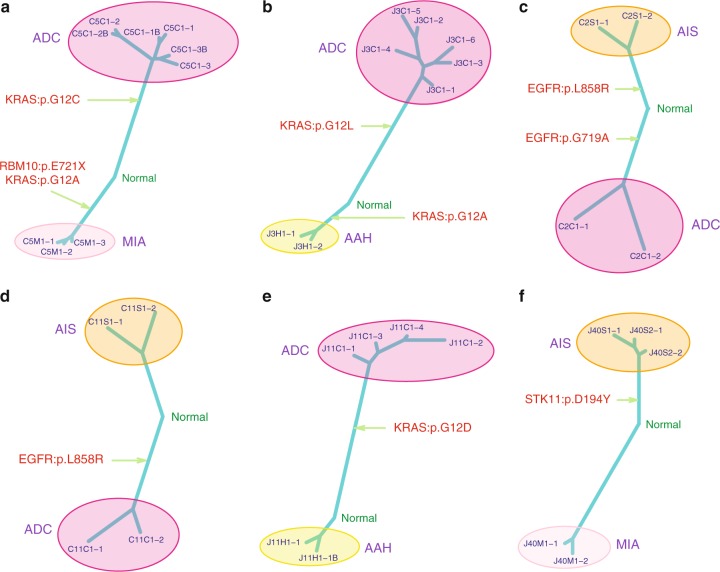
Representative phylogenetic trees of multifocal IPNs. **a–f** Phylogenetic trees were generated from all SNVs by using the Wagner parsimony method in “phangorn” package. Known cancer gene mutations are mapped to the trunks and branches as indicated. Trunk and branch lengths are proportional to the numbers of mutations acquired on the corresponding trunks or branchs

There were 39 patients with multifocal IPNs in this cohort, including 22 patients with more than one histologic stage of IPNs (Supplementary Data 1 and 6). These patients provided a unique opportunity to decipher the genetic constraints underlying the carcinogenesis of lung adenocarcinomas, as multiple lesions share identical genetic background and relative exposure history. Overall, the results were similar to those from the cohort as a whole with later-stage IPNs having higher TMB (Supplementary Fig. 11a), more AI events (Supplementary Fig. 11b) and higher proportion of clonal mutations (Supplementary Fig. 11c) compared to early-stage IPNs from the same patients. Interestingly, distinct cancer gene mutations were detected in different IPNs (Fig. 5a–f). On the other hand, some cancer genes demonstrated distinct mutations across different IPNs within the same patients. For example, although no mutations were shared between a MIA lesion and an ADC lesion from patient C5, implying these were two independent primary tumors, a *KRAS* p.G12A mutation was identified in an MIA and a p.G12C mutation in an ADC (Fig. 5a). Similarly, a *KRAS* p.G12A mutation and a *KRAS* p.G12L mutation were detected in an AAH lesion and an ADC lesion, respectively from patient J3 (Fig. 5b). The same phenomenon was also observed for *EGFR* mutations in patient C2 (Fig. 5c). These findings are reminiscent of heterogeneity studies in renal cell carcinoma, where different mutations in the same cancer genes were identified in different regions within the same tumor implying convergent evolution^29^. Taken together, these results suggest the possibility that even with an identical genetic background and environmental exposure, carcinogenesis of multiple primary tumors can be driven by distinct molecular events in different tumors, with possible genetic constraints around certain genes or pathways that are pivotal for carcinogenesis in certain patients.

## Discussion

The characterization of IPNs through multi-region WES in our study provided molecular evidence supporting the proposed model of early carcinogenesis of lung adenocarcinoma from AAH to AIS, MIA, and ADC, and revealed evidence supporting progressive genomic evolution at the single nucleotide level accompanied by macroevolution at the transition from AAH to AIS and from AIS to MIA, possibly associated with SCNA and AI, respectively (Supplementary Fig. 12). Meanwhile, we observed substantial inter-patient heterogeneity at each histologic stage. For example, although TMB is significantly higher in ADC than AAH as a group, some AAH lesions demonstrated higher TMB than many ADC lesions (Fig. 1a, Supplementary Figs. 4 and 10). Therefore, characterizing larger cohorts of AAH, AIS, MIA, and ADC lesions is necessary to address the inter-patient heterogeneity and to more robustly decipher common genomic evolutionary patterns from preneoplasia to invasive lung cancer. In addition, the current diagnosis of AAH, AIS, MIA, and ADC was based on morphologic assessment, which may not fully reflect the underlying biology of these lesions. In future studies, more definitive endpoints such as postsurgical recurrence and overall survival should be integrated with molecular landscape to define the molecular subtypes of IPNs and assess their prognostic values.

Determining whether pulmonary nodules are malignant or benign is critical for appropriate management of IPNs. However, it is challenging, particularly for ground glass opacity (GGO)-predominant IPNs, due to low yield of biopsy in such lesions^30^. Multiple algorithms incorporating clinical and radiologic features have been proposed to deduce cancer probability of IPNs, but the accuracy of these algorithms is still in question^30,31^. We tested a commonly used and recently validated algorithm^32,33^ in the IPNs of our cohort. Higher cancer risk scores were observed in later-stage IPNs (Supplementary Fig. 13), confirming the predictive value of this algorithm. However, there was substantial overlap between different histologic stages, highlighting the limitation of such algorithms. Since morphologic staging may not be optimal to reflect the biology of these IPNs, we tested whether cancer recurrence, a more clinically meaningful endpoint, was associated with certain genomic features. We did not detect significant associations between recurrence and TMB, abundance of AI events, proportion of clonal mutations, or APOBEC enrichment scores (Supplementary Data 7) in this cohort of patients. Of note, the follow up was short (median 12.4 months ranging from 0 to 42.6 months) and only four recurrences (three histologically confirmed recurrences and one suspected recurrence with brain metastasis) have occurred (Supplementary Data 1). Analyses of larger cohorts of IPNs with longer follow up are needed to identify potential molecular markers to select high-risk IPNs.

Intra-tumor heterogeneity (ITH) could provide diverse genetic and epigenetic elements to foster tumor evolution and the ITH architecture may evolve with neoplastic progression^34,35^. Evolution of ITH architecture can follow a subclonal diversification model, where tumor ITH becomes progressively more heterogeneous in more advanced diseases driven by acquisition of subclonal genomic events in different cell populations along with disease progression. Previous studies have shown higher level of ITH complexity in later-stage NSCLC, consistent with this model during progression from early stage to advanced NSCLC^11,12,36^. In interesting contrast, the data from current study revealed higher proportion of subclonal mutations (Fig. 3b) and branch mutations (Supplementary Fig. 9) in early-stage IPNs than those of advanced stages, suggesting a clonal sweep model with selective outgrowth of fit subclones during initiation and early progression of lung preneoplasia. These observations imply that neoplastic evolution is a dynamic process that may change along with neoplastic progression and follow different models at different stages. Furthermore, varying evolutional processes in different IPNs of each histologic stage were observed, further highlighting the substantial inter-patient heterogeneity even at the preneoplastic and preinvasive stages.

Our results have revealed evidence supporting genomic evolution with neoplastic progression from preneoplasia to invasive lung adenocarcinoma. However, as all patients in this study were from Japan and China, future studies using IPNs from Western patient populations are warranted to validate whether these findings are broadly applicable. In addition, a major caveat of the current study is that these analyses were based on resected lesions, which offered a single molecular snapshot of the evolutionary process of IPNs. There is an assumption of a linear model of evolution from AAH to AIS, MIA and ADC. However, whether all AAH eventually transform to AIS, MIA, or ADC and whether every ADC follows the linear evolutionary trajectory from AAH to AIS, then MIA and eventually ADC is unknown. These questions cannot be addressed by the analyses conducted in a single resected specimen. For example, one alternative explanation for the lack of reduction in subclonal TMB in later-stage IPNs (Fig. 3a and Supplementary Fig. 8) is the “winner effect”, where only IPNs with high mutational burden (both clonal and subclonal) would eventually transform to IPNs of advanced stages. Deciphering how the genomic landscape evolves over time with neoplastic progression and how these changes associate with patient outcomes requires longitudinal biopsies over the course of disease progression from lung preneoplasia, which is impractical in general clinical practice. Conversely, lung cancer prevention trials applying longitudinal biopsies such as IMPRINT-Lung (NCT03634241) may provide a unique opportunity to examine the temporal changes in molecular features with neoplastic progression.

## Methods

### Patients and tissue processing

Resected specimens were collected from patients presenting with IPNs, who underwent resection at Zhejiang Cancer Hospital (China) and Nagasaki Hospital (Japan) from 2014 to 2017. None of these patients received preoperative chemotherapy or radiotherapy (Supplementary Data 1). Rigorous pathology quality control was applied and all samples were subjected to central pathology review at MD Anderson prior to further analyses. A “grid” approach (Supplementary Fig. 1) was used to collect tissues from multiple regions within each IPN. Manual macrodissection was applied to ensure a minimum of 40% diseased (atypical or malignant) cells in each multi-region sample before DNA extraction. Samples with lower disease content were excluded from further analyses. DNA from normal lung tissue (≥2 cm from tumor margin, morphologically negative for malignant cells assessed by two lung cancer pathologists independently) from the same patients was used as germ line DNA control. Written informed consent was obtained from all patients involved. The study was approved by the Institutional Review Boards (IRB) at MD Anderson Cancer Center, Zhejiang Cancer Hospital and Nagasaki University Graduate School of Biomedical Sciences.

### Whole-exome sequencing

DNA was extracted using the QIAamp DNA FFPE Tissue Kit (QIAGEN) and the resulting genomic DNA was sheared into 300–400 bp segments and subjected to library preparation for whole-exome sequencing using KAPA library prep (Kapa Biosystems) with the Agilent SureSelect Human All Exon V4 kit according to the manufacturer’s instructions. 76nt paired-end multiplex sequencing of DNA samples was performed on the Illumina HiSeq 2500 sequencing platform.

### SNV and indel calling from whole-exome sequencing

Sequencing reads were mapped to the human reference sequence GRCh37 (hg19) using the Burrows-Wheeler Aligner (BWA) using default parameters^37^. Duplicate reads were marked using Picard 1.67 followed by realignment around known indels and base quality recalibration was performed using GATK version 3.7^38^. Somatic mutation calls were performed using Mutect 1.1.4, allowing at least 0.05 variant allele frequency in the tumor sample and up to a maximum of 0.01 allele frequency in normal sample, with sequencing depth of at least 20× in tumor and 10× in normal samples, as well as mutation LOD score >10. The passed variants were further filtered through validation using other somatic variant callers including somaticsniper, freebayes, vardict, MuSE, and only mutations detected by at least two somatic variant callers were subjected to further analyses. Small indels of cancer genes were detected by pindel and further filtered with total tumor reads >15 and total normal reads >6; at least 4 reads supporting the indel with a minimum allele frequency of 0.05 in tumor and maximum 0.01 in normal, to obtain a more confident set of somatic variants. Then forced callings were performed based on shared mutation loci detected by Mutect in multiple regions from one lesion. The final list of somatic SNVs and indels was then annotated by multiple databases using Annovar and filtered by dbsnp129.

### Quality assessment of FFPE samples

To evaluate the potential sequencing artifacts derived from FFPE samples in this study, mutation calls from TCGA LUAD cohort were downloaded^14^, the ratio of C > T/G > A transitions was calculated and compared to that in our cohort of IPNs.

### Mutational signature analysis

“DeconstructSigs” package was applied to extract top mutational signatures based on non-negative matrix factorization (NMF) and model selection to deconstruct mutational processes present in each lesion. The curated mutational signature sets are based on combined Alexandrov and COSMIC signatures (AC1-30)^19^ used in “YAPSA” package. To avoid over-fitting, we only applied signature analysis to IPNs with at least 100 unique SNVs. APOBEC-mediated mutational processes were defined as previously described. In brief, APOBEC enrichment scores reflecting the strength of mutagenesis at the TCW (where W is either A or T) motif was determined for all mutations in each lesion^11^.

### Copy number and allelic imbalance analysis

Disease and matched germ line DNA were used to obtain tumor-specific (somatic) copy number changes by varscan2^39^ from WES data, the log2 ratios of disease versus germ line DNA reads were calculated for each tumor region after adjusting for the total mapped reads in that tumor region, then segmented by the circular binary segmentation (CBS) algorithm^40^. The GISTIC2 algorithm^41^ was applied to the segmented copy number profiles to identify significant aberrations of broad and focal events. Similar approach has been taken to estimate copy number status at the gene level. Log ratios were subjected to segmentation using the “DNAcopy” package, and then segment data were processed using the “CNTools” package to generate segmented DNA copy number matrix. To determine the somatic copy number aberrations of cancer genes in this cohort, oncogenes known to be activated by amplification and tumor suppressor genes known to be inactivated by deletion were examined. A threshold of log2 ratio (IPN versus germ line DNA) >2 or <−2 was used to screen for copy number gains or copy number losses, respectively. Manual inspection on IGV was conducted to review all segments containing candidate genes in each sample to confirm chromosomal gains or losses.

The regions of genomic allelic imbalance were predicted by hapLOHseq based on the probabilities for each heterozygous genotype residing in a region present allelic imbalance^24^, which leverages the allele-specific read counts and capturing signals among multiple sites jointly at haplotype level.

### Subclonal analysis

Tumor purity was estimated using ABSOLUTE^42^ and ASCAT^43^. The cancer cell fraction (CCF) and mutant allele copy number for each SNV was inferred using pyclone 12.3^44^ following the modified method described previously^45^. In brief, PyClone implements a Dirichlet process clustering model that simultaneously estimates the distribution of the cellular prevalence for each mutation. Copy numbers of somatic mutations were inferred by integrating integer copy numbers determined by ASCAT on single sample basis. The outputs were cellular prevalence value distributions per SNV estimated from Markov-chain Monte Carlo (MCMC) sampling. The median value of the MCMC sampling-derived distribution was used as a representative cellular prevalence for each mutation. A given mutation was classified as “clonal” if the 95% confidence interval of CCF overlapped 1 and “subclonal” otherwise.

### Phylogenetic analysis

Using binary matrix based on the presence and absence of somatic mutations across samples, we first calculated the genetic distances between samples using the hamming distance, then applying neighbor joining algorithm and wagner parsimony method from the APE and phangorn package^46^ to infer phylogenetic relationships between tumor sectors for each patient.

### Cancer risk prediction

Brock University cancer prediction equation as below was used to predict the probability of cancer in each lesion^32,33^.

$${\begin{array}{l}{\mathrm{Log}}\,{\mathrm{odds}} = \left( {0.0287\left( {{\mathrm{Age}} - 62} \right)} \right) + {\mathrm{Sex}} + {\mathrm{Family}}\,{\mathrm{History}}\,{\mathrm{Lung}}\,{\mathrm{Cancer}} + {\mathrm{Emphysema}}\\ - \left( {5.3854\left( {\left( {{\mathrm{Nodule}}\,{\mathrm{size}}/10} \right)^{ - 0.5}-1.58113883} \right) + {\mathrm{Nodule}}\,{\mathrm{type}}} \right.\\ + {\mathrm{Nodule}}\,{\mathrm{Upper}}\,{\mathrm{Lung}} - 0.0824\left( {{\mathrm{Nodule}}\,{\mathrm{count}} - 4} \right)\\ + {\mathrm{Spiculation}} - 6.7892\end{array}}$$

$${{\mathrm{Cancer}}\,{\mathrm{probability}} = 100\left( {{\mathrm{e}}^{({\mathrm{Logodds}})}/\left( {1 + {\mathrm{e}}^{({\mathrm{Logodds}})}} \right)} \right)}$$

### Statistical analyses

Kruskal–Wallis H test was applied to assess the association between mutational burdens, proportion of clonal mutations and trunk mutations, APOBEC signature enrichment, abundance of allelic imbalance events, risk scores between IPNs from different histologic stages. Tukey’s test was used for comparing mutational burden between each stage pairs. One-sided Wilcoxon rank-sum test was used for the comparison between the groups of smokers and non-smokers. All statistical analysis was performed using R.

### Reporting summary

Further information on research design is available in the Nature Research Reporting Summary linked to this article.

## Supplementary information

Supplementary Information

Description of Additional Supplementary Files

Supplementary Data 1

Supplementary Data 2

Supplementary Data 3

Supplementary Data 4

Supplementary Data 5

Supplementary Data 6

Supplementary Data 7

Reporting Summary

## Data Availability

The data from whole-exome sequencing has been deposited at European Genome-phenome Archive (EGA), which is hosted by The European Bioinformatics Institute (EBI) and the Centre for Genomic Regulation (CRG) under the accession code: EGAS00001004960 [https://www.ebi.ac.uk/ega/datasets/EGAD00001004960]. Further information about EGA is available at https://ega-archive.org. All other data may be found within the main manuscript or Supplementary Information or available from the authors upon request.
