# Author Correction: Multi-region exome sequencing reveals genomic evolution from preneoplasia to lung adenocarcinoma

**DOI:** 10.1038/s41467-021-23163-3

**Published:** 2021-05-12

**Authors:** Xin Hu, Junya Fujimoto, Lisha Ying, Junya Fukuoka, Kazuto Ashizawa, Wenyong Sun, Alexandre Reuben, Chi-Wan Chow, Nicholas McGranahan, Runzhe Chen, Jinlin Hu, Myrna C. Godoy, Kazuhiro Tabata, Kishio Kuroda, Lei Shi, Jun Li, Carmen Behrens, Edwin Roger Parra, Latasha D. Little, Curtis Gumbs, Xizeng Mao, Xingzhi Song, Samantha Tippen, Rebecca L. Thornton, Humam Kadara, Paul Scheet, Emily Roarty, Edwin Justin Ostrin, Xu Wang, Brett W. Carter, Mara B. Antonoff, Jianhua Zhang, Ara A. Vaporciyan, Harvey Pass, Stephen G. Swisher, John V. Heymach, J. Jack Lee, Ignacio I. Wistuba, Waun Ki Hong, P. Andrew Futreal, Dan Su, Jianjun Zhang

**Affiliations:** 1 Department of Genomic Medicine, The University of Texas MD Anderson Cancer Center, Houston, TX 77030 USA; 2 Department of Translational Molecular Pathology, The University of Texas MD Anderson Cancer Center, Houston, TX 77030 USA; 3 Institute of Cancer Research and Basic Medical Sciences of Chinese Academy of Sciences, Cancer Hospital of University of Chinese Academy of Sciences, Zhejiang Cancer Hospital & Key Laboratory Diagnosis and Treatment Technology on Thoracic Oncology of Zhejiang Province, 310022 Hangzhou, China; 4 Department of Pathology, Nagasaki University Graduate School of Biomedical Sciences, 8528523 Nagasaki, Japan; 5 Department of Clinical Oncology, Nagasaki University Graduate School of Biomedical Sciences, 8528523 Nagasaki, Japan; 6 Department of Pathology, Institute of Cancer Research and Basic Medical Sciences of Chinese Academy of Sciences, Cancer Hospital of University of Chinese Academy of Sciences, Zhejiang Cancer Hospital, 310022 Hangzhou, China; 7 Department of Thoracic/Head and Neck Medical Oncology, The University of Texas MD Anderson Cancer Center, Houston, TX 77030 USA; 8 Cancer Research United Kingdom-University College London Lung Cancer Centre of Excellence, London, WC1E6BT UK; 9 Department of Diagnostic Radiology, The University of Texas MD Anderson Cancer Center, Houston, TX 77030 USA; 10 Department of Radiology, Institute of Cancer Research and Basic Medical Sciences of Chinese Academy of Sciences, Cancer Hospital of University of Chinese Academy of Sciences, Zhejiang Cancer Hospital, 310022 Hangzhou, China; 11 Department of Epidemiology, The University of Texas MD Anderson Cancer Center, Houston, TX 77030 USA; 12 Department of General Internal Medicine, The University of Texas MD Anderson Cancer Center, Houston, TX 77030 USA; 13 Department of Thoracic and Cardiovascular Surgery, The University of Texas MD Anderson Cancer Center, Houston, TX 77030 USA; 14 Department of Cardiothoracic Surgery, New York University Langone Medical Center, New York, NY 10016 USA; 15 Department of Biostatistics, The University of Texas MD Anderson Cancer Center, Houston, TX 77030 USA

**Keywords:** Cancer genomics, Non-small-cell lung cancer

Correction to: *Nature Communications* 10.1038/s41467-019-10877-8, published online 5 July 2019.

The original version of this Article contained an error in the Data Availability Statement. The accession code indicated ‘EGAS00001004960’ and should have read ‘ EGAS00001003439’. This has been corrected in both PDF and HTML versions of the Article.

