# Supplementary material for: Multi-region exome sequencing reveals genomic evolution from preneoplasia to lung adenocarcinoma
Source: Nat Commun. 2019 Jul 5;10:2978. doi: 10.1038/s41467-019-10877-8 (PMC6611767; doi:10.1038/s41467-019-10877-8)
Supplement: Supplementary file 1 — Supplementary Information [file 41467_2019_10877_MOESM1_ESM.pdf]

Suppplemental Information

Multi-region exome sequencing reveals genomic evolution from preneoplasia to lung adenocarcinoma

Hu, et al.

a

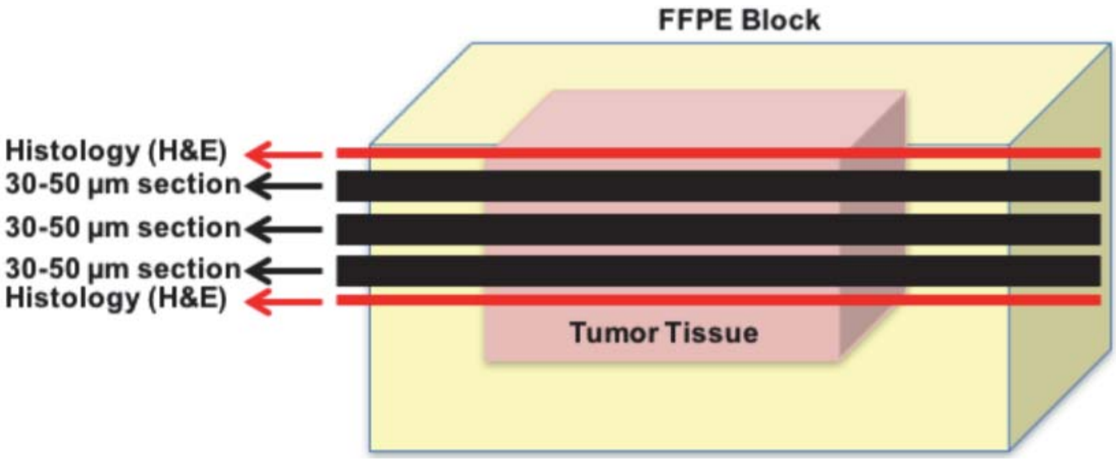

b

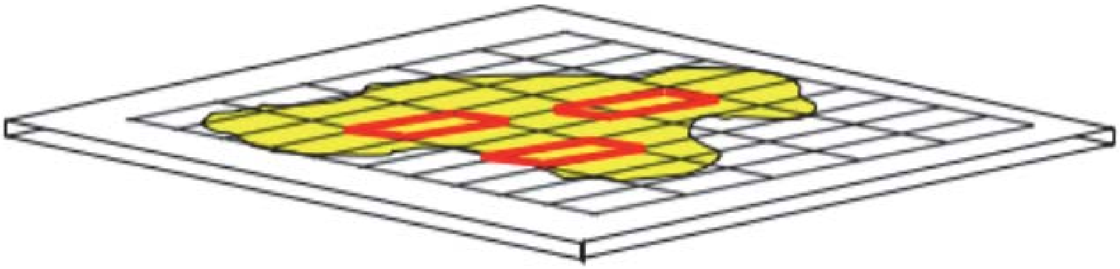

c

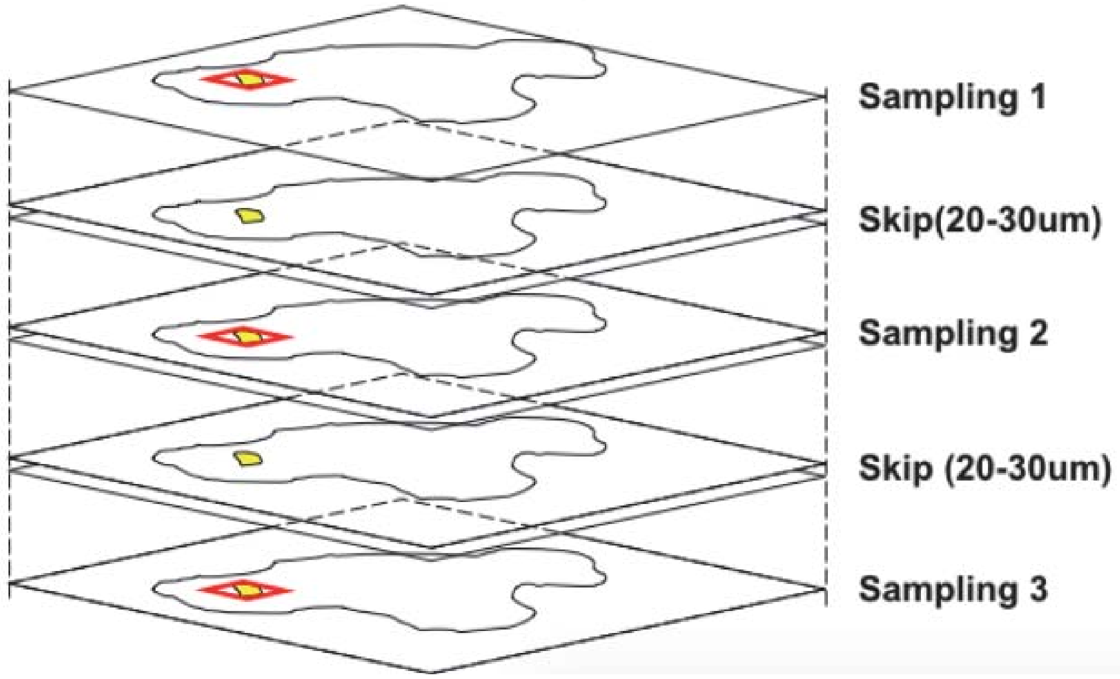

Supplementary Figure 1. Schema of tissue "grid" methodology for multi-region sampling. (a) Illustration of a 30-50 µm thick section from FFPE block. Tissue is consecutively sectioned for histology evaluation (H&E), 30-50 µm section, followed by 5 µm histology sections for HE assessment. **(b)** A 50µm thick section from a large lesion like surgically resected lung adenocarcinoma. A grid is superimposed between H&E and thick sections to identify and select multiple spatially separated 3 x 3 mm regions (*red squares*) to be dissected for DNA extraction using Biobank Card system. **(c)** A 30µm thick section from a small lesion like AAH. FFPE block was sectioned into multiple layers to obtain multiple spatially separated (at least 20-30µm apart) 3x3 mm tissue pieces (*red squares*) to be dissected for DNA extraction using Biobank Card system.

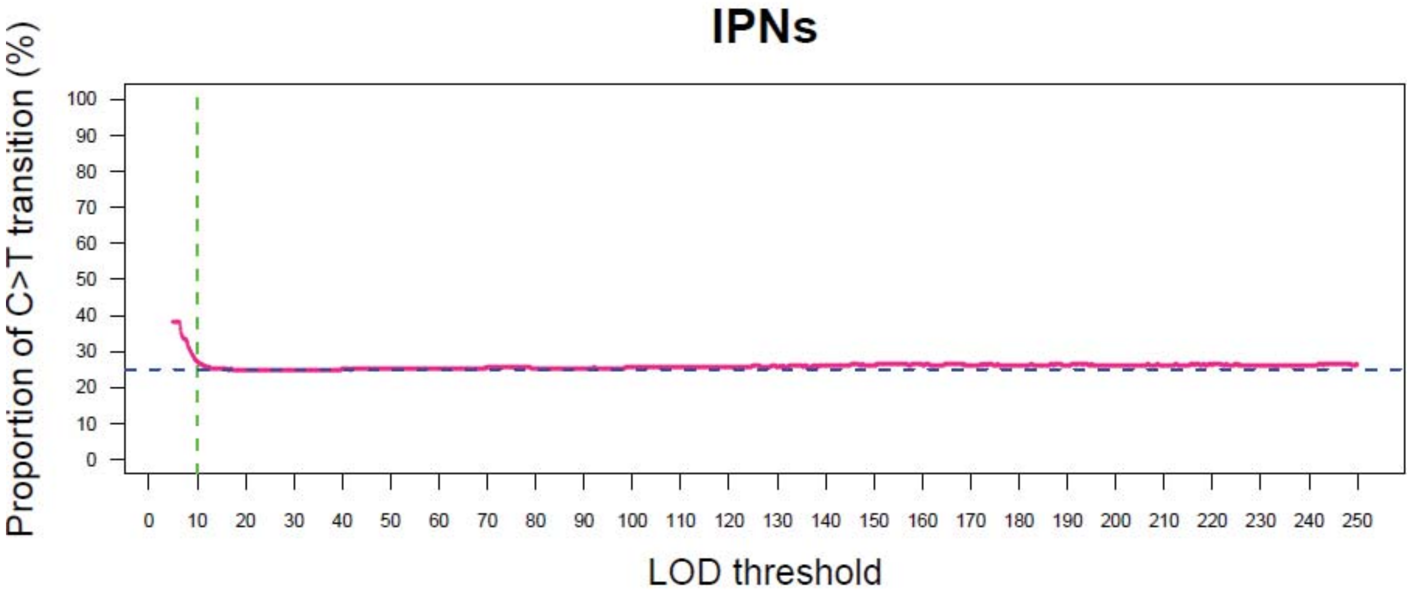

Supplementary Figure 2. The distribution of the proportion of C>T/G>A transitions by LOD scores. X-axis shows the log odds (LOD) scores of mutation calls and Y-axis represents the proportion of C>T/G>A transitions among all mutations of any given LOD threshold.

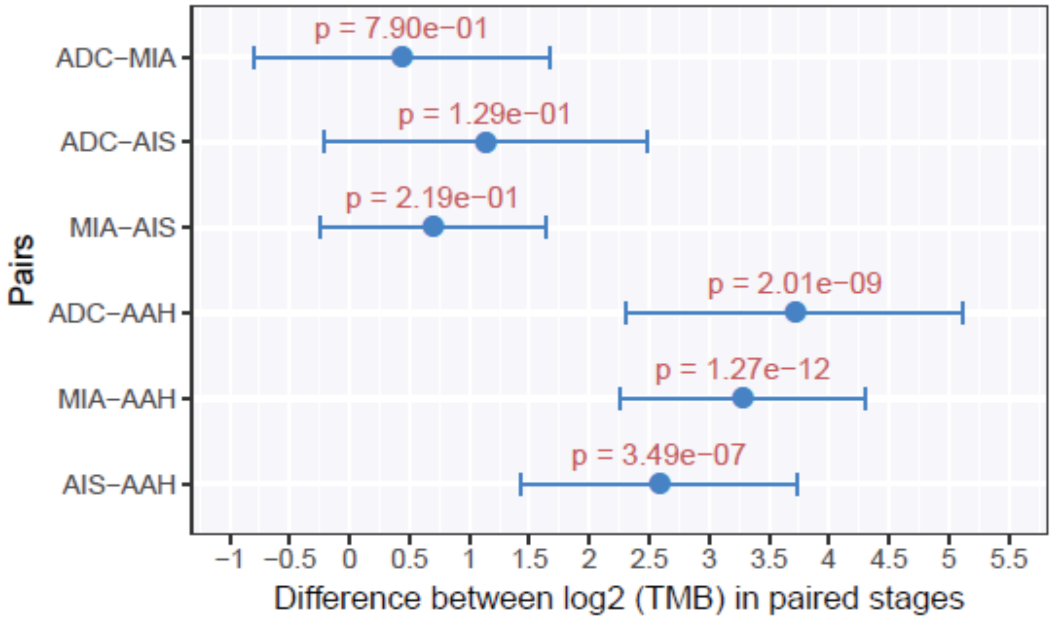

Supplementary Figure 3. Pairwise comparisons of TMB in IPNs of different histologic stages. The solid blue dots represent the difference of the average mutation burden (log2 transformed) between each stage pairs. The error bars represent Standard Error (SE) with 95% confident interval in each comparison. Tukey's test was used for comparing mutation burden between each stage pairs.

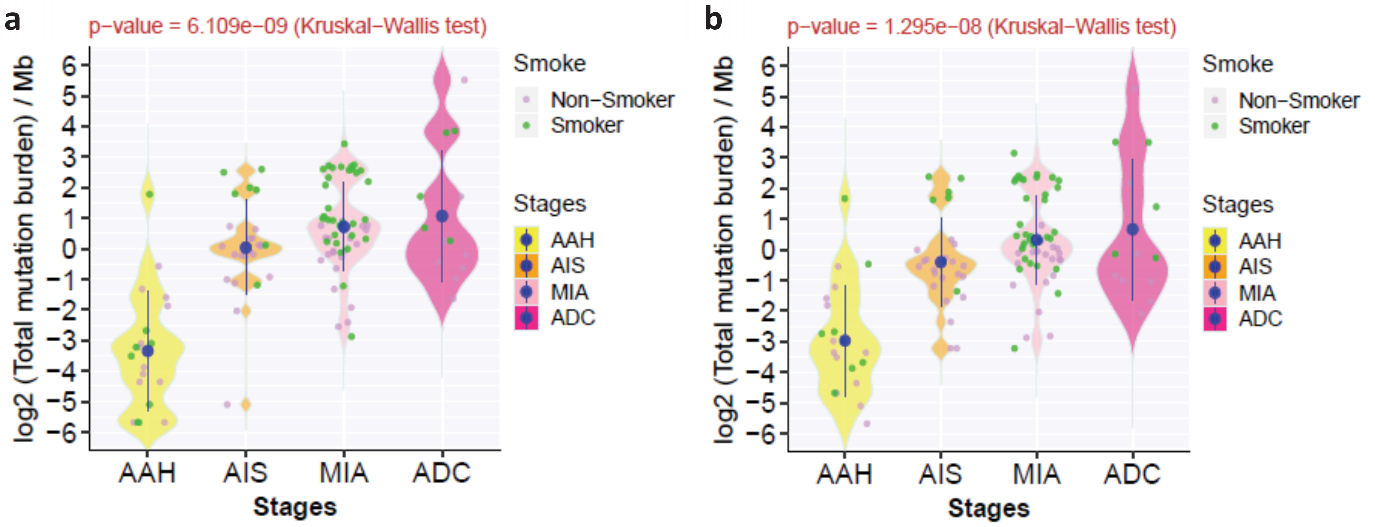

Supplementary Figure 4. The mutation burden of IPNs of different stages after filtering out potential FFPE artifacts by removing private subclonal mutations (a) or removing all C>T/G>A transitions (b). Each green (smoker) or purple (non-smoker) dot represents the mutation burden per megabase (MB) in each IPN and the solid blue dots represent the mean mutation burden of all lesions in each histologic stage. Kruskal-Wallis H test was used for comparing mutation burden between all stages.

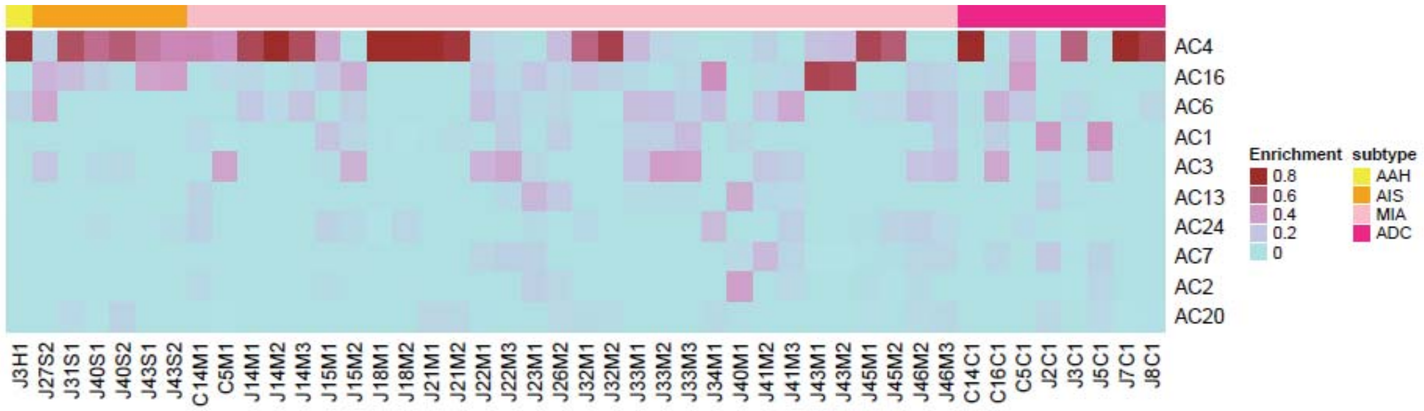

Supplementary Figure 5. The top 10 mutational signatures. The Alexandrov-COSMIC (AC) mutation signatures were derived from mutations in each IPN. Only IPNs with a minimum of 100 unique SNVs were included in the mutational signature analysis. The heat map represents the weight of each AC mutational signature in each IPN.

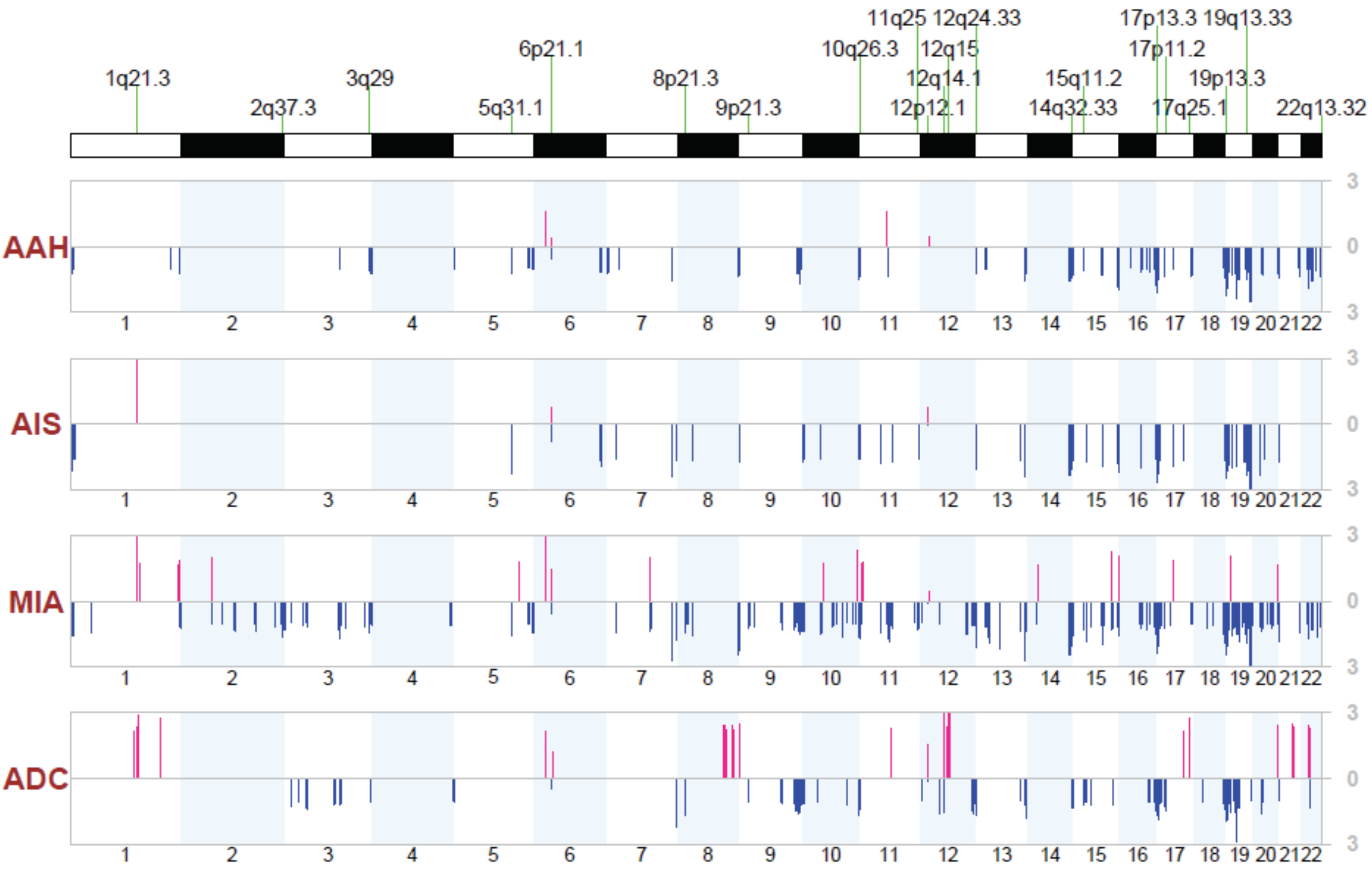

Supplementary Figure 6. Recurrent focal copy-number aberrations in IPNs grouped by each stage. The red lines represent genomic regions with significant copy number gains and the blue lines represent genomic regions with significant copy number losses with false discovery rate q-value <5e-06 across all the samples in each stage.

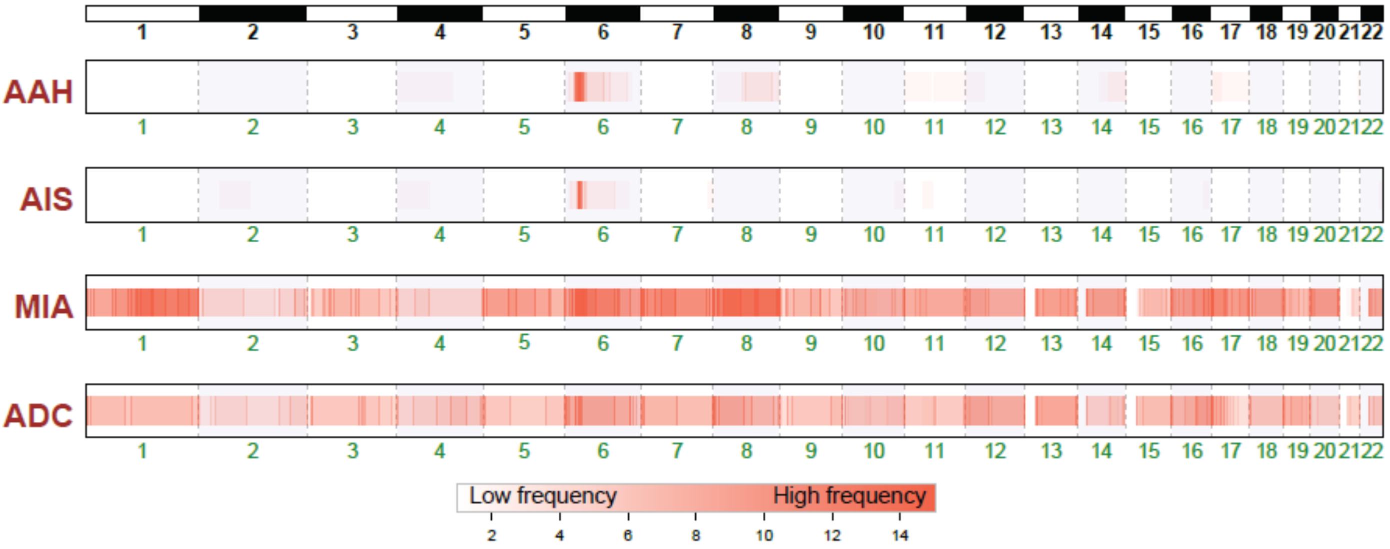

Supplementary Figure 7. Allelic imbalance (AI) events across the genome grouped by stage. The red shade represents the chromosomal regions with AI events identified. The level of transparency in red shade represents the frequency of AI events detected at specific chromosomal regions.

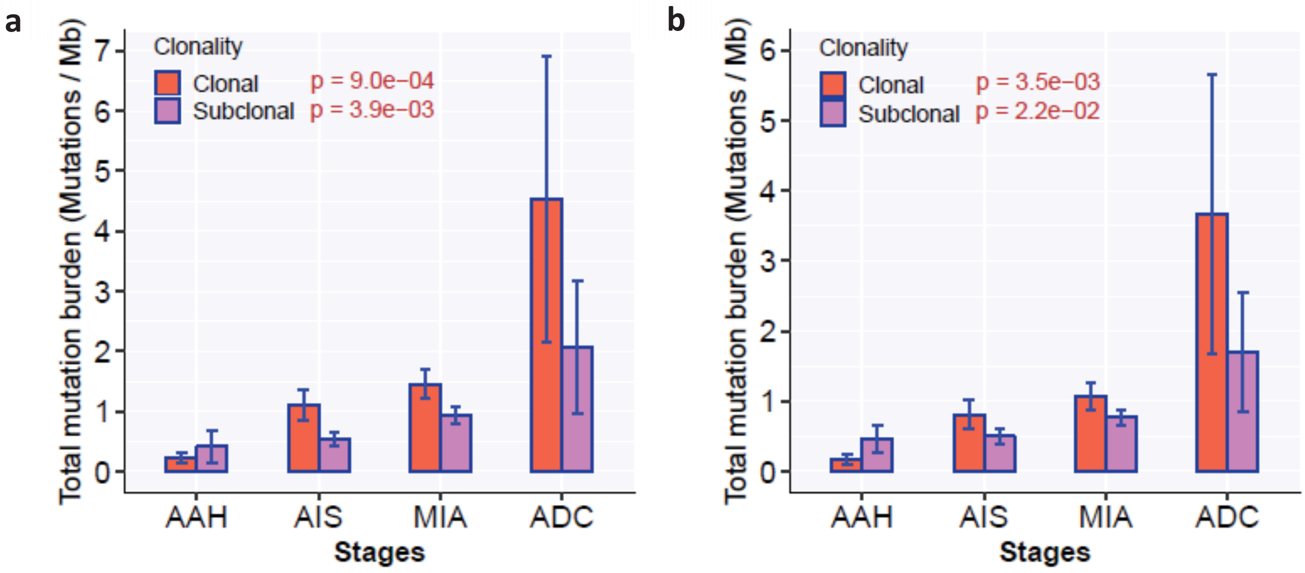

Supplementary Figure 8. The clonal mutation burden and subclonal mutation burden in IPNs of different histologic stages after filtering out potential FFPE artifacts by removing all private subclonal mutations (a) or removing all C>T/G>A transitions (b). The mean clonal mutation burden (orange) and subclonal mutation burden (purple) in AAH, AIS, MIA and ADC are shown with 95% confidence interval as error bars. Kruskal-Wallis H test was used to compare clonal and subclonal mutation burdens between all stages, respectively. Only IPNs with a minimum of 10 SNVs were included for this analysis.

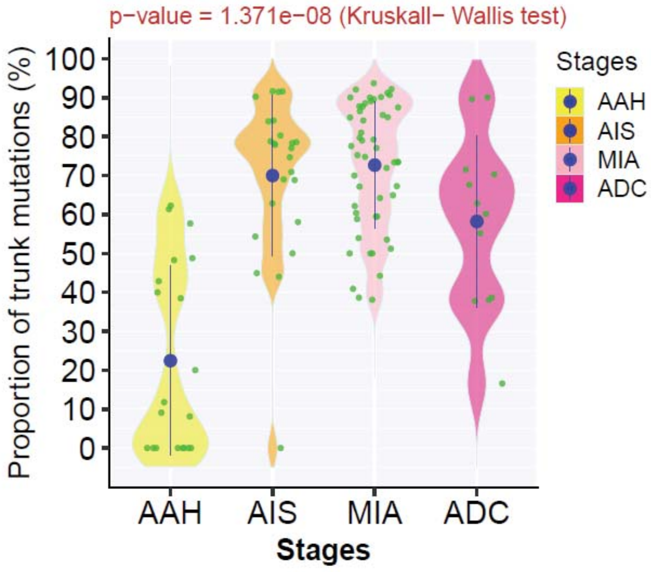

Supplementary Figure 9. The proportion of trunk mutations in IPNs of different stages. The mean proportion of trunk mutations in AAH, AIS, MIA and ADC is shown with 95% confidence interval as error bars. The difference between all stages was assessed by Kruskal-Wallis H test. For this analysis, IPNs with sequencing data from 2-6 regions were included. For IPNs with 2 or 3 regions sequenced, trunk mutations were defined as mutations detected in all regions sequenced. For the IPNs with 4 regions sequenced (2 MIA and 3 ADC) and 6 regions sequenced (7 ADC), trunk mutations were defined as mutations detected in at least 3 regions of the IPN.

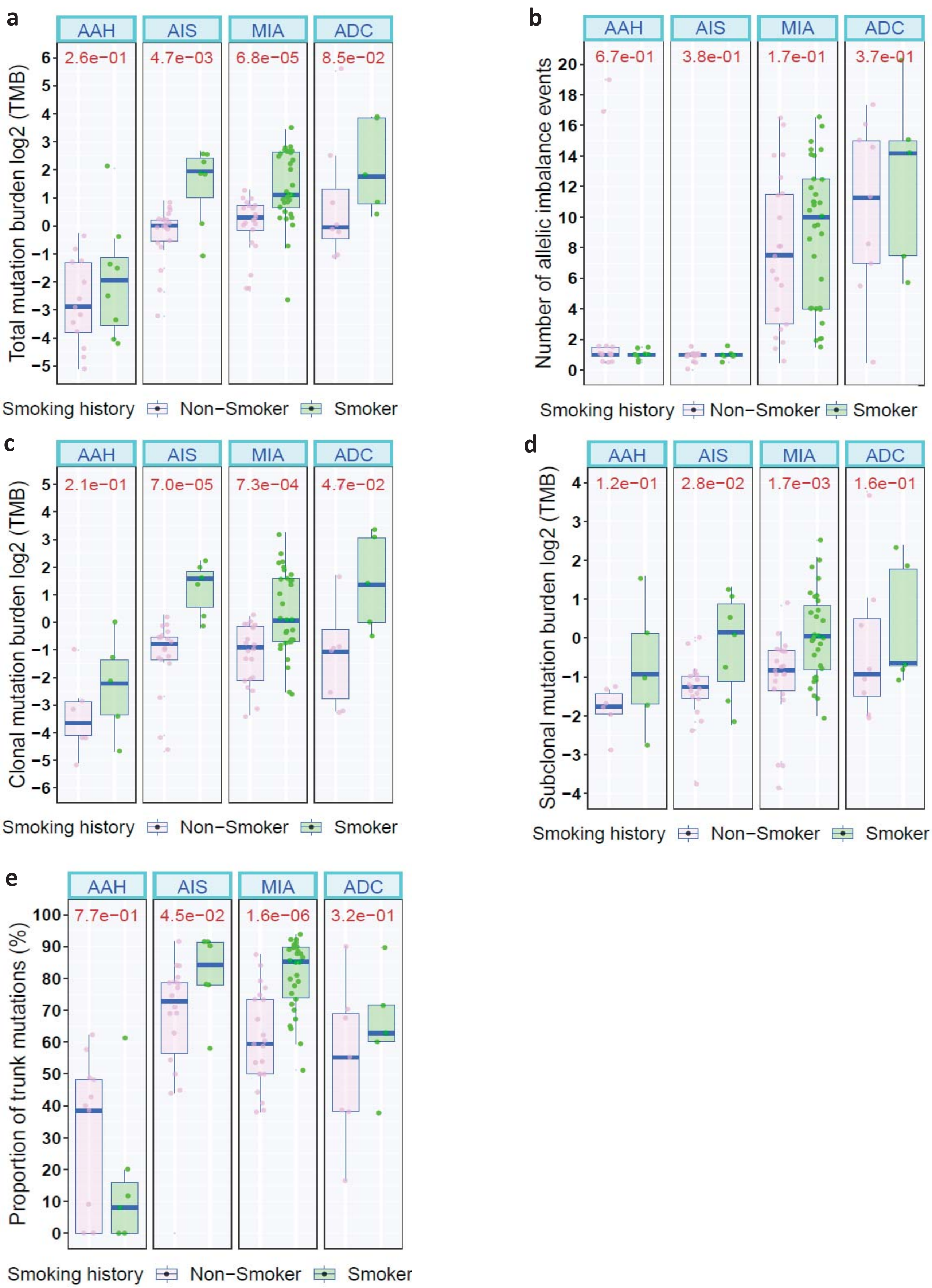
a
AAH
AIS
MIA
ADC
2.6e−01
4.7e−03
6.8e−05
8.5e−02
Total mutation burden log2 (TMB)
Smoking history
Non−Smoker
Smoker
b
AAH
AIS
MIA
ADC
6.7e−01
3.8e−01
1.7e−01
3.7e−01
Number of allelic imbalance events
Smoking history
Non−Smoker
Smoker
c
AAH
AIS
MIA
ADC
2.1e−01
7.0e−05
7.3e−04
4.7e−02
Clonal mutation burden log2 (TMB)
Smoking history
Non−Smoker
Smoker
d
AAH
AIS
MIA
ADC
1.2e−01
2.8e−02
1.7e−03
1.6e−01
Subclonal mutation burden log2 (TMB)
Smoking history
Non−Smoker
Smoker
e
AAH
AIS
MIA
ADC
7.7e−01
4.5e−02
1.6e−06
3.2e−01
Proportion of trunk mutations (%)
Smoking history
Non−Smoker
Smoker

Supplementary Figure 10. Genomic landscape of IPNs of different stages in smokers versus non-smokers for total mutation burden (a), number of allelic imbalance events (b), clonal mutation burden (c), subclonal mutation burden (d), and proportion of trunk mutations (e). The purple dots represent data from non-smokers and green dots represent data from smokers. The solid blue lines represent the median of each histologic stage with 95% confidence interval as error bars. A one-sided Wilcoxon Rank Sum test was used for comparisons between non-smokers and smokers. Subclonal analysis was applied to IPNs with a minimum of 10 SNVs. For trunk mutation analysis, IPNs with sequencing data from 2-6 regions were included. For IPNs with 2 or 3 regions sequenced, trunk mutations were defined as mutations detected in all regions sequenced. For the IPNs with 4 regions sequenced (2 MIA and 3 ADC) and 6 regions sequenced (7 ADC), trunk mutations were defined as mutations detected in at least 3 regions of the IPN.

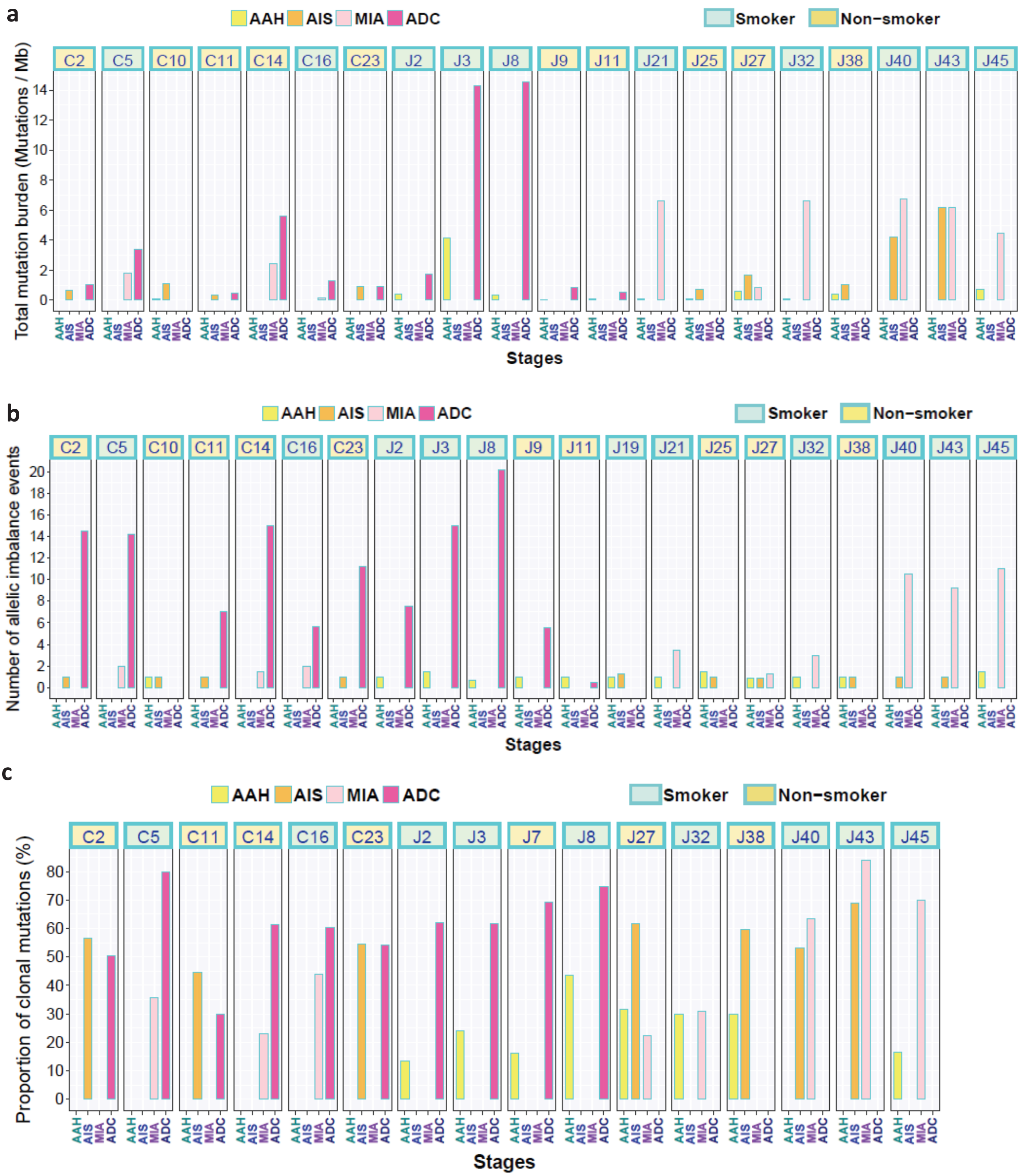

Supplementary Figure 11. Genomic features of IPNs in patients with multifocal diseases for total mutation burden (a), number of allelic imbalance events (b) and proportion of clonal mutations (c). Histologic stages of IPNs and smoking status of each patient are indicated in different colors. Subclonal analysis was applied to IPNs with a minimum of 10 SNVs.

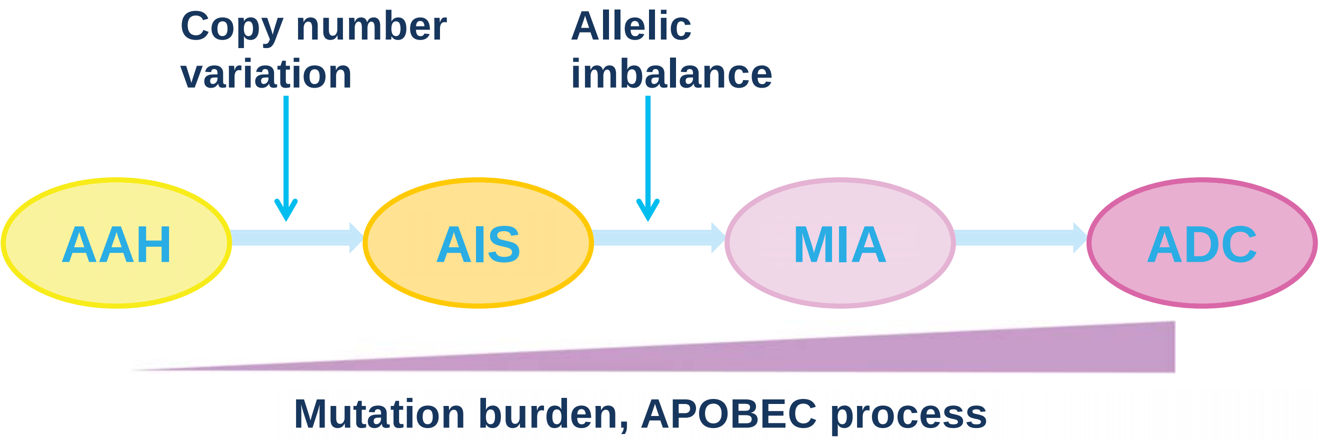

Supplementary Figure 12. Genomic evolution from preneoplasia to invasive lung adenocarcinoma. Neoplastic progression from AAH to AIS, MIA and ADC is associated with a progressive increase in tumor mutation burden, APOBEC processes, while somatic copy number changes and allelic imbalance are demarcated that may occur at the transition from AAH to AIS and from AIS to MIA respectively.

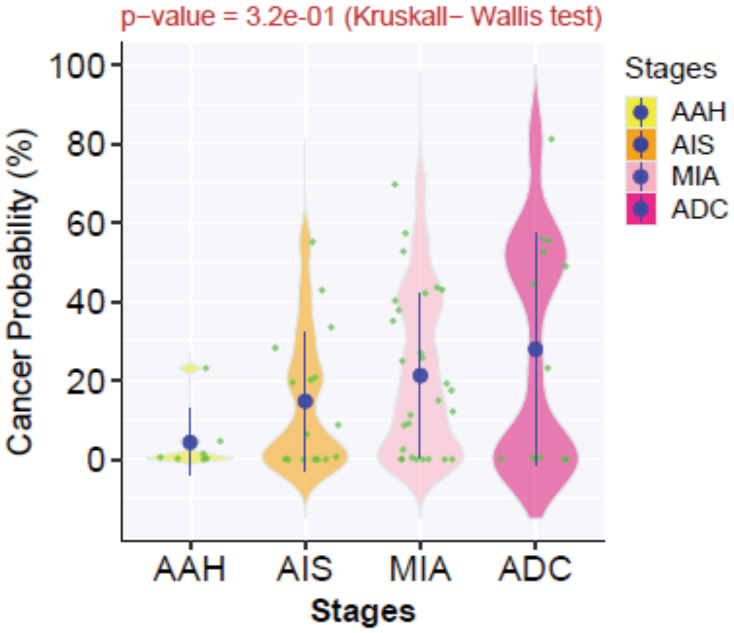

Supplementary Figure 13. Predicted cancer risks in IPNs of different histologic stages. The mean cancer probability estimated using Brock University algorithm for IPNs of each histologic stage was labeled by solid blue dots. Each green dot represents predicted cancer probability in each IPN. The difference between all stages was assessed by Kruskal-Wallis H test.
