# Supplementary material for: Multi-region exome sequencing reveals genomic evolution from preneoplasia to lung adenocarcinoma
Source: Nat Commun. 2019 Jul 5;10:2978. doi: 10.1038/s41467-019-10877-8 (PMC6611767; doi:10.1038/s41467-019-10877-8)
Supplement: Supplementary file 2 — Description of Additional Supplementary Files [file 41467_2019_10877_MOESM2_ESM.pdf]

Description of Additional Supplementary Files

File Name: Supplementary Data 1
Description: Patient characteristics.

File Name: Supplementary Data 2
Description: List of single nucleotide variants (SNVs).

File Name: Supplementary Data 3
Description: List of samples with high proportion of C>T/G>A transitions.

File Name: Supplementary Data 4
Description: List of cancer gene mutations and copy number variations.

File Name: Supplementary Data 5
Description: Canonical EGFR mutations.

File Name: Supplementary Data 6
Description: List of patients with multifocal IPNs.

File Name: Supplementary Data 7
Description: Comparison of genomic features of patients with recurrence to those without recurrence.
