# Supplementary material for: Multi-region exome sequencing reveals genomic evolution from preneoplasia to lung adenocarcinoma
Source: Nat Commun. 2019 Jul 5;10:2978. doi: 10.1038/s41467-019-10877-8 (PMC6611767; doi:10.1038/s41467-019-10877-8)
Supplement: Supplementary file 10 — Reporting Summary [file 41467_2019_10877_MOESM10_ESM.pdf]

nature research

Corresponding author(s): Jianjun Zhang

Last updated by author(s): Apr 20, 2019

Reporting Summary

Nature Research wishes to improve the reproducibility of the work that we publish. This form provides structure for consistency and transparency in reporting. For further information on Nature Research policies, see Authors & Referees and the Editorial Policy Checklist.

Statistics

For all statistical analyses, confirm that the following items are present in the figure legend, table legend, main text, or Methods section.

n/a	Confirmed	
☐	☒	The exact sample size (*n*) for each experimental group/condition, given as a discrete number and unit of measurement
☐	☒	A statement on whether measurements were taken from distinct samples or whether the same sample was measured repeatedly
☐	☒	The statistical test(s) used AND whether they are one- or two-sided<br>*Only common tests should be described solely by name; describe more complex techniques in the Methods section.*
☐	☒	A description of all covariates tested
☐	☒	A description of any assumptions or corrections, such as tests of normality and adjustment for multiple comparisons
☐	☒	A full description of the statistical parameters including central tendency (e.g. means) or other basic estimates (e.g. regression coefficient) AND variation (e.g. standard deviation) or associated estimates of uncertainty (e.g. confidence intervals)
☐	☒	For null hypothesis testing, the test statistic (e.g. *F*, *t*, *r*) with confidence intervals, effect sizes, degrees of freedom and *P* value noted<br>*Give P values as exact values whenever suitable.*
☐	☒	For Bayesian analysis, information on the choice of priors and Markov chain Monte Carlo settings
☐	☒	For hierarchical and complex designs, identification of the appropriate level for tests and full reporting of outcomes
☐	☒	Estimates of effect sizes (e.g. Cohen's *d*, Pearson's *r*), indicating how they were calculated

Our web collection on statistics for biologists contains articles on many of the points above.

Software and code

Policy information about availability of computer code

Data collection	No software was used for data collection.
Data analysis	Mutect1.1.4, varscan2, freebayes, vardict, MuSE, pindel, GISTIC2, IGV, ASCAT, pyclone. All statistic analyses were performed in R.

For manuscripts utilizing custom algorithms or software that are central to the research but not yet described in published literature, software must be made available to editors/reviewers. We strongly encourage code deposition in a community repository (e.g. GitHub). See the Nature Research guidelines for submitting code & software for further information.

Data

Policy information about availability of data

All manuscripts must include a data availability statement. This statement should provide the following information, where applicable:

- Accession codes, unique identifiers, or web links for publicly available datasets
- A list of figures that have associated raw data
- A description of any restrictions on data availability

We are submitting BAM files of exome-seq data for all the samples to EGA, with EGA accession number (EGAS00001003439)

Field-specific reporting

Please select the one below that is the best fit for your research. If you are not sure, read the appropriate sections before making your selection.

☒ Life sciences ☐ Behavioural & social sciences ☐ Ecological, evolutionary & environmental sciences

For a reference copy of the document with all sections, see nature.com/documents/nr-reporting-summary-flat.pdf

Life sciences study design

All studies must disclose on these points even when the disclosure is negative.

Sample size	Sample size was not determined before the study. The results of all patients/samples that fulfilled the inclusion criteria described in the method session were included.
Data exclusions	We excluded samples that failed sequencing.
Replication	No replication was applied to any individual sample. But multiple regions from the same lesions were sequenced.
Randomization	NA. All eligible patients/samples were included.
Blinding	NA

Reporting for specific materials, systems and methods

We require information from authors about some types of materials, experimental systems and methods used in many studies. Here, indicate whether each material, system or method listed is relevant to your study. If you are not sure if a list item applies to your research, read the appropriate section before selecting a response.

Materials & experimental systems

n/a	Involved in the study	
☒	☐	Antibodies
☒	☐	Eukaryotic cell lines
☒	☐	Palaeontology
☒	☐	Animals and other organisms
☐	☒	Human research participants
☒	☐	Clinical data

Methods

n/a	Involved in the study	
☒	☐	ChIP-seq
☒	☐	Flow cytometry
☒	☐	MRI-based neuroimaging

Human research participants

Policy information about studies involving human research participants

Population characteristics	All patients were from China and Japan, with median age of 70 (range 44-83) and the ratio of male to female is 32:21. All patients presented as pulmonary nodules and treated with upfront surgical resection. None of the patients received neoadjuvant treatment.
Recruitment	No patients were recruited particularly for this study. Patients were treated as standard of care and resected specimens were analyzed.
Ethics oversight	Written informed consent was obtained from all patients involved. The study was approved by institutional review board (IRB).

Note that full information on the approval of the study protocol must also be provided in the manuscript.
